# Adaptive optimal output regulation for wheel-legged robot Ollie: A data-driven approach

**DOI:** 10.3389/fnbot.2022.1102259

**Published:** 2023-01-12

**Authors:** Jingfan Zhang, Zhaoxiang Li, Shuai Wang, Yuan Dai, Ruirui Zhang, Jie Lai, Dongsheng Zhang, Ke Chen, Jie Hu, Weinan Gao, Jianshi Tang, Yu Zheng

**Affiliations:** ^1^Tencent Robotics X, Tencent Holdings, Shenzhen, Guangdong, China; ^2^School of Computer Science, Yangtze University, Jingzhou, Hubei, China; ^3^State Key Laboratory of Synthetical Automation for Process Industries, Northeastern University, Shenyang, China; ^4^School of Integrated Circuits, Tsinghua University, Beijing, China

**Keywords:** optimal control, output regulation, adaptive control, data-driven control, wheel-legged robot

## Abstract

The dynamics of a robot may vary during operation due to both internal and external factors, such as non-ideal motor characteristics and unmodeled loads, which would lead to control performance deterioration and even instability. In this paper, the adaptive optimal output regulation (AOOR)-based controller is designed for the wheel-legged robot Ollie to deal with the possible model uncertainties and disturbances in a data-driven approach. We test the AOOR-based controller by forcing the robot to stand still, which is a conventional index to judge the balance controller for two-wheel robots. By online training with small data, the resultant AOOR achieves the optimality of the control performance and stabilizes the robot within a small displacement in rich experiments with different working conditions. Finally, the robot further balances a rolling cylindrical bottle on its top with the balance control using the AOOR, but it fails with the initial controller. Experimental results demonstrate that the AOOR-based controller shows the effectiveness and high robustness with model uncertainties and external disturbances.

## 1. Introduction

The control performance deterioration on real robots is often blamed on the inaccurate model. First, it is hard to model the robot accurately and to obtain the precise model parameters. In addition, the robotic system may vary during operation, so the inherent model also varies. Furthermore, some environmental changes in terrains, loads, etc. are also reflected as variations in the robot dynamics. The model issue is troublesome in general and could be hazardous for highly dynamic and statically unstable robots, such as two-wheel and wheel-legged robots. For example, the model-based optimal control is designed for the ballbot (Lauwers et al., 2006), but the model should be updated when a load, such as an arm, is equipped (Nagarajan et al., 2012). In addition, for the wheel-legged robot Ascento (Klemm et al., 2020), the optimality of the model-based control may be hard to be ensured when the working condition changes. Furthermore, for the two-wheel robot Segway, the robustness of the balance control may not overcome working condition variations, which lead to serious injuries (Ashurst and Wagner, 2015).

The issue on the model accuracy motivates the application of non-model-based or data-driven control algorithms, such as data-driven MPC (Willems et al., 2005; Jianwang and Ramirez-Mendoza, 2022) and adaptive dynamic programming (ADP) (Jiang and Jiang, 2012a, 2013, 2017; Bian and Jiang, 2016). For both of them, an optimal stabilizing controller can be trained using small data without system identification, which makes online implementation possible.

Particularly, the ADP consists of the policy iteration (PI) method and the value iteration (VI) method. The ADP was explored in our previous researches and proved to be useful, especially for the balance control of configuration changeable and statically unstable robots (Cui et al., 2021; Zhang et al., 2022). By the PI, the initial control should be stabilizing but not necessarily be optimal, and the training results in an approximated optimal controller (Jiang and Jiang, 2012a,b). This property is suitable when the configuration of the robot changes. By the VI, the initial control can be arbitrary and even manual or unstable. The training by the VI results in an initial optimal controller at the cost of longer iterations to converge (Bian and Jiang, 2016, 2022). This property is significant for statically unstable robots, because the training can start without any controller design. In the most recent study (Qasem et al., 2021), the proposed hybrid iteration combines the advantages of PI and VI. Moreover, the controller iteration in the ADP is robust to the noisy data in sense of data with the bounded noise leading to a controller bounded in a neighborhood of the nominal optimal solution (Pang et al., 2022). Compared with reinforcement learning applications on the balance control of two-wheel robots (majorly in simulation) (Raudys and Šubonienė, 2020; Guo et al., 2021), the ADP requires small data and the stability can be proved theoretically, which enables the training on real robots directly and removes the troublesome sim-to-real gap.

Besides the model uncertainty, another issue may affect the control performance is unmodeled disturbances on real robots. The disturbance may be caused by any mismatch between the ideal simulation and the real robot, such as the terrain changes, uncertain frictions and slight offsets of robot states, which are hard to be sourced and analyzed completely. For wheel-legged robots, a common consequence of these disturbances is the steady-state error. In our previous works, although the controller is stabilizing, the robot cannot stand still without extra manual adjustment, which is mentioned as an unsolved problem in Zhang et al. (2022). This phenomenon is common in real-robot experiments of two-wheel and wheel-legged robots (Jung and Kim, 2008; Huang et al., 2012; Zafar et al., 2019; Zhang et al., 2019; Zhou et al., 2021), but it is unnoticed if the asymptotic stability is not required strictly.

In the existing literature on wheel-legged robots, the steady-state error is eliminated by the centroidal adjustment in Zhou et al. (2019) and by arm acceleration control in Raza et al. (2021). In addition to the case-by-case solutions to the disturbance, a more systematic approach is to apply output regulation on selected outputs (Isidori and Byrnes, 1990; Huang, 2004). In Wang et al. (2021), the output regulation is applied to force the robot to stand still using the cart-pole model where the disturbance is estimated by an observer.

To overcome the both aforementioned issues by model uncertainties and unmodeled disturbances, a novel solution is the adaptive optimal output regulation (AOOR) (Gao and Jiang, 2016, 2022). Similar to the standard ADP, the training can be conducted on real robots directly, and the system identification is not needed. In the author's previous researches, the optimal output regulation has been applied to semi-autonomous vehicles (Huang et al., 2019) and connected autonomous vehicles (Gao et al., 2016, 2019; Gao and Jiang, 2017). However, there is no application on balance augmentation for wheel-legged robots.

**Figure 1:**
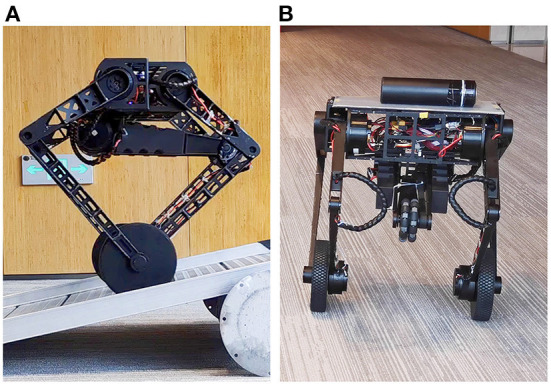
The wheel-legged robot Ollie. Many more maneuvers in action and experimental data are shown in the accompanying video. **(A)** Train the controller on a slope. **(B)** Test the controller to balance a cup.

In this paper, we design the AOOR-based controller on the wheel-legged robot Ollie (Figure 1) to achieve non-model-based optimal control and regulate it to stand still in various working conditions. Compared with our previous works (Cui et al., 2021; Wang et al., 2021; Zhang et al., 2022), the contributions in this paper are concluded as follows.

- In Cui et al. (2021) and Zhang et al. (2022), the robot is balanced but cannot stand still, which was stated as a limitation of the ADP algorithm. In this paper, the problem is solved by further regulating the velocity of the robot to zero in a systematic approach.
- The robot was forced to stand still when it is at the minimum height (Wang et al., 2021) based on the cart-pole model, but this model-based method cannot keep the robot standing still when robot configuration or the environment changes. In this paper, the data-driven approach based on the ADP is applied to regulate the robot adaptively.
- In addition to solving the problems in the scenarios of our previous works, more challenging scenarios are invoked to test the generality of the proposed control algorithm.

The structure of the remaining paper is as follows. In Section 2.1, the robot system and the control framework are introduced briefly, with the problem statement of the control given in Section 2.3. In Section 3, the main controller design based on the AOOR is provided with its algorithm. In Section 4, the experiment results on the training process and the related testing process using the AOOR-based controller are demonstrated, and an interesting application is also shown. Finally, conclusions and several feasible future studies are given in Section 5.

## 2. Robotic system and control framework

### 2.1. Introduction to the robotic system

**Figure 2:**
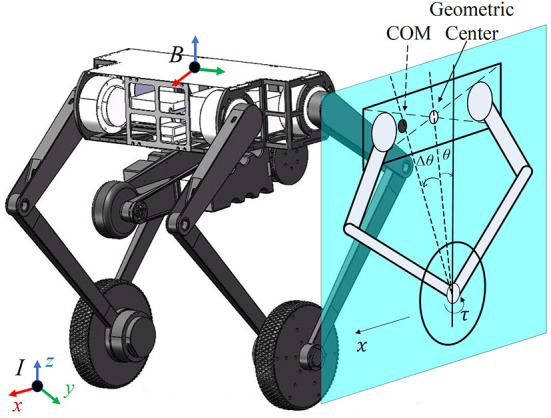
Mechanical design of the robot Ollie and simplified model on the sagittal plane, where the geometric center and the CoM are marked in a hollow circle and a filled circle, respectively. The actual pitch angle $\bar{\theta }$ is the subtraction of the measured value θ and an offset Δθ, i.e., $\bar{\theta }=\theta -\Delta \theta$. The displacement of the robot in the x direction is denoted as *x* with its velocity ẋ. The wheel is actuated by the motor torque τ.

The robot we used in this paper is the wheel-legged robot Ollie. This robot was self-developed by Tencent Robotics X and was first released in both the plenary talk and the research paper (Wang et al., 2021) at ICRA 2021. Ollie (mechanical model shown in Figure 2) is designed using eight driving motors: four motors on the hips, two motors on the tail and two motors on the wheels. The five-bar linkage mechanism is used so that each leg is actuated by two motors on the base, without actuators on the knees. Based on the mechanical design, each five-bar leg is able to move along the *x* and *z*-axis in the leg plane, which keeps perpendicular to the robot base. Except for two-wheel balancing, the robot has the ability to switch flexibly between two-wheel and three-wheel modes. Moreover, the attitude of the base can be adjusted by changing the height of each leg, i.e., distance between the center of the wheel and the base in the leg plane. With an optimal mechanical design of legs, the robot can stride over obstacles, jump, and flip, demonstrating its strong ability to realize dynamic movements (Ackerman, 2021). The total weight of Ollie is around 15 kg. The height of the robot is between 0.33 and 0.7 m.

### 2.2. Robotic control framework

**Figure 3:**
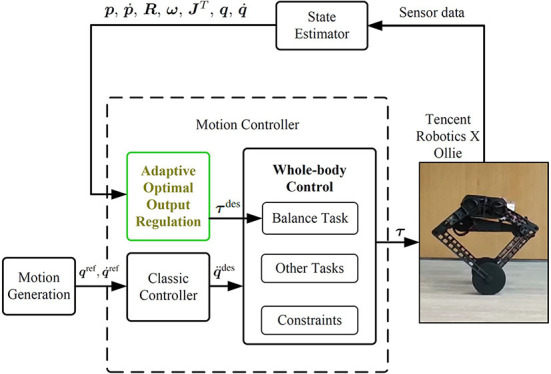
Control framework of the robot system.

In order to keep Ollie moving with agile and flexible body poses at the same time of wheel balancing, the whole-body control (WBC) is used. The robotic control framework is shown in Figure 3. See details of the whole-body dynamics model and optimization formulation in the authors' previous publication (Zhang et al., 2022).

Before the WBC module, an individual controller is designed to generate the reference torques **τ**^des^ for the balancing task due to the nonminimum-phase property of the wheel-legged robot. Conventionally, references of the balancing task in the WBC are usually generated by controller design based on a simplified model (Klemm et al., 2020; Murtaza et al., 2020) or by a manual tuning controller (Zambella et al., 2019). To overcome the aforementioned model uncertainties and inaccuracy of model parameters and external disturbances (formally stated in Section 2.3), the AOOR is used to calculate balancing task references.

Other task references, such as attitudes and height of the robot, are obtained from the motion generation and classic controllers.

### 2.3. Problem statement

The problem mentioned in Zhang et al. (2022) is that it is hard for such a wheel-legged robot to keep standing completely still in different working conditions. In order to make the robot stand still, the center of mass (CoM) and the middle of wheels should be vertical. Therefore, the accurate estimation of the CoM position is crucial for model-based controllers, but the deviation from the expected position is inevitable in practice due to the nonuniform mass distribution and the assembly errors. This is presented in the sagittal plane in Figure 2, where the CoM of the floating base is assumed to be consistent with the geometric center but they are different in practice.

In the balancing task, the pitch angle $\bar{\theta }$, pitch angular velocity $\dot{\bar{\theta }}$ and the robot's linear velocity $\dot{\bar{x}}$ are regulated to zero by controlling the motor torque τ at the wheel. The balancing system can be expressed in the linear form


(1) $$\begin{array}{l} \dot{\bar{\zeta }}=A\bar{\zeta }+B\tau , \end{array}$$

where $\bar{\zeta }={\left[\bar{\theta }\;\dot{\bar{\theta }}\;\dot{\bar{x}}\right]}^{T}$, ***A*** ∈ ℝ^*n*×*n*^ and ***B*** ∈ ℝ^*n*×*m*^ (*n* = 3, *m* = 1). It must be highlighted that, due to the uncertain factors of the robotic system, it is hard to model an accurate expression of ***A*** and ***B***, and they are not used in this paper.

Particularly, considering the deviation of the CoM, the real pitch angle is the subtraction of the measured value θ and an offset Δθ, i.e., $\bar{\theta }=\theta -\Delta \theta$ (Note that subtraction is used to keep consistent with the formulation in Section 3), while the real value and the measured value are equal for the other two states ($\dot{\bar{\theta }}=\dot{\theta }$, $\dot{\bar{x}}=ẋ$). Then, the dynamic model (Equation 1) is equivalent to


(2) $$\begin{array}{l} \dot{\zeta }=A\zeta +B\tau +D, \end{array}$$

where $\zeta ={\left[\theta ,\dot{\theta },ẋ\right]}^{T}$, ***D*** ∈ ℝ^*n*×*q*^ (*q* = 1) and ***D*** = ***A***[−Δ, θ, 0, 0]^*T*^.

In addition, to stop the robot at a fixed position, ẋ is chosen as the output to be regulated to zero, and the error is


(3) $$\begin{array}{l} e=C\bar{\zeta }=C\zeta , \end{array}$$

where ***C*** = [0 0 1].

In this paper, we consider the balancing task in the WBC framework with the system described in Equations (2) and (3) with the unknown parameters ***A***, ***B***, and Δθ. The objective is to stabilize the system by optimal control adaptively and to regulate the output to zero.

## 3. AOOR-based balance control algorithm

One possible way to keep the wheel-legged robot standstill while balancing is to design a controller based on the AOOR. A similar data-driven optimal output regulation for linear systems with exogenous disturbances is proposed in Gao and Jiang (2016), but the controller design based on the similar data-driven method on the balance augmentation of wheel-legged robots is not trivial. In this section, we design the AOOR-based controller for the particular application on the balancing task of wheel-legged robots.

### 3.1. Model-based optimal output regulation

The standard state feedback control of the system (Equation 1) is


(4) $$\begin{array}{l} \tau =-K\bar{\zeta } \end{array}$$

when (***A***, ***B***) is stabilizable, where ***K*** ∈ ℝ^*n*×1^ is a stabilizing feedback gain. The control law can be rewritten in the equivalent form for the system (Equation 2) as


(5) $$\begin{array}{l} \tau =-K\zeta +L \end{array}$$

where *L* = ***KZ*** + *T* with ***Z*** = [Δθ 0 0]^*T*^. In the control law (Equation 5), the first term regulates the state **ζ**, and the second term compensates the uncertainty ***D***. Then, the original system (Equation 1) is asymptotically stable ($\underset{t\to \infty }{\lim}\bar{\zeta }=0$) if the unknown ***Z*** and *T* are solvable by the regulator equation (see the proof in Huang, 2004):


(6) $$\begin{array}{l} \begin{array}{c} 0=AZ+BT+D,\\ 0=CZ. \end{array} \end{array}$$

Due to the specific underactuated dynamics of the wheel-legged robot, there exists a unique solution of the regulation equation. It is self-evident because the steady-state must be **ζ** = [Δθ, 0, 0] for the standstill condition, and the torque is then fixed. However, for other types of robots, there may exist multiple solutions, and an optimization problem can be designed to solve the optimal solution (Gao and Jiang, 2016).

In this paper, the optimality is represented by solving the following constrained optimization problem for the state feedback control.


(7) $$\begin{array}{l} \begin{array}{c} \underset{\tau }{\min}\int_{0}^{\infty }\left({\bar{\zeta }}^{T}Q\bar{\zeta }+R\tau^{2}\right)dt\\ \text{subject to (Equation 1)} \end{array} \end{array}$$

where ***Q*** ∈ ℝ^*n*×*n*^ is positive-definite and *R* ∈ ℝ is positive. The optimal solution is represented by the optimal feedback gain


(8) $$\begin{array}{l} K^{*}=-R^{-1}B^{T}P^{*} \end{array}$$

where the positive-definite P^*^ is the solution of the algebraic Riccati equation (ARE) (Lewis et al., 1995).

### 3.2. Data-driven optimal output regulation

In the data-driven approach, the main objective is to remove the dependency on the model parameter ***A***, ***B*** and ***D***, and determine the optimal solution (K^*^, P^*^) and (***Z***, *T*).

To determine the optimal feedback gain K^*^, by utilizing the collected data **ζ**(*t*) and τ(*t*), the optimization problem (Equation 7) is solved iteratively. Particularly, the dependency on ***A*** is removed by the Lyapunov equation


(9) $$\begin{array}{l} -K_{j}^{T}RK_{j}-Q=A_{j}^{T}P_{j}+P_{j}A_{j} \end{array}$$

where *j* is the index of iteration. Meanwhile, the dependency on ***B*** is removed by the gain iteration


(10) $$\begin{array}{l} K_{j+1}=R^{-1}B^{T}P_{j}. \end{array}$$

In addition, ***D*** is set as a variable to be determined in each iteration. When ***K*** and ***P*** converge, the results can be proved as the optimal solutions K^*^ and P^*^ (Kleinman, 1968).

To determine the regulation parameters (***Z***, *T*), a basis of ***Z*** is defined as $\hat{Z}={\left[1\;0\;0\right]}^{T}$ such that $Z=\alpha \hat{Z}$ with a scalar α and the second equation in the regulator (Equation 6) is satisfied. Notice that the construction of the basis of ***Z*** is slightly different from Gao and Jiang (2016) in this particular application on Ollie. Then, by defining a Sylvester map *S*:ℝ^*n*^↦ℝ^*n*^ by *S*(***Z***) = −***AZ***, the first equation in the regulation equation is rewritten as


(11) $$\begin{array}{l} S\left(Z\right)\equiv \alpha S\left(\hat{Z}\right)=BT+D=P^{*}K^{*T}RT+D \end{array}$$

where ***B*** is represented by Equation (10) using K^*^ and P^*^ from the previous step, and ***D*** is solved in the previous step.

Then, if $S\left(\hat{Z}\right)$ is available, the pair (***Z***, *T*) can be computed by solving the following linear equation


(12) $$\begin{array}{l} \left[\begin{array}{ccc} S\left(\hat{Z}\right) & 0 & -P^{*}K^{*T}R\\ \hat{Z} & -I & 0 \end{array}\right]\left[\begin{array}{c} \alpha \\ Z\\ T \end{array}\right]=\left[\begin{array}{c} D\\ 0 \end{array}\right]. \end{array}$$

Finally, by setting *L* = K^*^Z + *T*, the optimal output regulation law in Equation (5) is attained.

The proposed AOOR is provided in Algorithm 1, with the related matrices defined in Equations (13) and (14), where the notation vec() denotes the vectorization, and ⊗ denotes the Kronecker product. It is suggested to check the conditioning number of **Ψ** and $\hat{\text{Ψ}}$ to avoid potential numerical problems caused by ill-conditioned matrix. The general idea of the proof follows from Gao and Jiang (2016), but the AOOR-based control is applied for the specific problem on the balance augmentation of wheel-legged robots. Due to the page limit, the detailed proof is omitted.


(13) $$\begin{array}{l} \begin{array}{c} \Lambda_{\zeta ,\zeta }={\left[\zeta ⊗\zeta {|}_{t_{0}}^{t_{1}},\zeta ⊗\zeta {|}_{t_{1}}^{t_{2}},\ldots ,\zeta ⊗\zeta {|}_{t_{s}}^{t_{s-1}}\right]}^{T}\\ \Sigma_{\zeta ,\zeta }={\left[\int_{t_{0}}^{t_{1}}\zeta ⊗\zeta dt,\int_{t_{1}}^{t_{2}}\zeta ⊗\zeta dt,\ldots ,\int_{t_{s-1}}^{t_{s}}\zeta ⊗\zeta dt\right]}^{T}\\ \Sigma_{\zeta ,\tau }={\left[\int_{t_{0}}^{t_{1}}\zeta ⊗\tau dt,\int_{t_{1}}^{t_{2}}\zeta ⊗\tau dt,\ldots ,\int_{t_{s-1}}^{t_{s}}\zeta ⊗\tau dt\right]}^{T}\\ \Sigma_{\zeta }={\left[\int_{t_{0}}^{t_{1}}\zeta dt,\int_{t_{1}}^{t_{2}}\zeta dt,\ldots ,\int_{t_{s}}^{t_{s-1}}\zeta dt\right]}^{T} \end{array} \end{array}$$

(14) $$\begin{array}{l} \begin{array}{c} \Lambda_{\hat{\zeta },\hat{\zeta }}={\left[\hat{\zeta }⊗\hat{\zeta }{|}_{t_{0}}^{t_{1}},\hat{\zeta }⊗\hat{\zeta }{|}_{t_{1}}^{t_{2}},\ldots ,\hat{\zeta }⊗\hat{\zeta }{|}_{t_{s}}^{t_{s-1}}\right]}^{T}\\ \Sigma_{\hat{\zeta },\hat{\zeta }}={\left[\int_{t_{0}}^{t_{1}}\hat{\zeta }⊗\hat{\zeta }dt,\int_{t_{1}}^{t_{2}}\hat{\zeta }⊗\hat{\zeta }dt,\ldots ,\int_{t_{s-1}}^{t_{s}}\hat{\zeta }⊗\hat{\zeta }dt\right]}^{T}\\ \Sigma_{\hat{\zeta },\tau }={\left[\int_{t_{0}}^{t_{1}}\hat{\zeta }⊗\tau dt,\int_{t_{1}}^{t_{2}}\hat{\zeta }⊗\tau dt,\ldots ,\int_{t_{s-1}}^{t_{s}}\hat{\zeta }⊗\tau dt\right]}^{T}\\ \Sigma_{\hat{\zeta }}={\left[\int_{t_{0}}^{t_{1}}\hat{\zeta }dt,\int_{t_{1}}^{t_{2}}\hat{\zeta }dt,\ldots ,\int_{t_{s}}^{t_{s-1}}\hat{\zeta }dt\right]}^{T} \end{array} \end{array}$$

**Algorithm 1:** Adaptive optimal output regulation.

1:	Apply the initial stabilizing control τ = −***K***_0_**ζ** + β with the exploration noise β(*t*),
	and collect data on [*t*_0_, *t*_*s*_].
2:	Compute the matrices in Equations (13) and (14).
3:	Ensure the rank condition holds.
	$\text{rank}\left(\left[\Sigma_{\zeta ,\zeta },\Sigma_{\zeta ,\tau },\Sigma_{\zeta }\right]\right)=\frac{n\left(n+1\right)}{2}+n\left(m+q\right)=12,$
	$\text{rank}\left(\left[\Sigma_{\hat{\zeta },\hat{\zeta }},\Sigma_{\hat{\zeta },\tau },\Sigma_{\hat{\zeta }}\right]\right)=\frac{n\left(n+1\right)}{2}+n\left(m+q\right)=12.$
4:	Set *j* = 0.
5:	**While** ||***P***_*j*+1_−***P***_*j*_|| < ϵ with some small ϵ
6:	Solve ***P***_*j*_, ***K***_*j*+1_ and ***D*** by
	$\text{Ψ}\left[\begin{array}{c} \text{vec}\left(P_{j}\right)\\ \text{vec}\left(K_{j+1}\right)\\ \text{vec}\left(D^{T}P_{j}\right) \end{array}\right]=\Phi$
	where
	$\text{Ψ}=\left[\Lambda_{\zeta ,\zeta },-2\Sigma_{\zeta ,\zeta }\left(I_{n}⊗K_{j}^{T}R\right)-2\Sigma_{\zeta ,\tau }\left(I_{n}⊗R\right),-2\Sigma_{\zeta ,\tau }\right]$
	$\Phi =-\Sigma_{\zeta ,\zeta }\text{vec}\left(Q+K_{j}^{T}RK_{j}\right).$
7:	**End while**
8:	Set $K^{*}=K_{j+1}$, $P^{*}=P_{j}$ as optimal solutions for Equation (7).
9:	Solve $S\left(\hat{Z}\right)$ by
	$\hat{\text{Ψ}}\left[\begin{array}{c} \text{vec}\left(P^{*}\right)\\ \text{vec}\left(K^{*}\right)\\ \text{vec}\left(\left(D^{T}-S\left(\hat{Z}\right)\right)P^{*}\right) \end{array}\right]=\hat{\Phi }$
	where
	$\hat{\text{Ψ}}=\left[\Lambda_{\hat{\zeta },\hat{\zeta }},-2\Sigma_{\hat{\zeta },\hat{\zeta }}\left(I_{n}⊗K^{*T}R\right)-2\Sigma_{\hat{\zeta },\tau }\left(I_{n}⊗R\right),-2\Sigma_{\hat{\zeta },\tau }\right]$
	$\hat{\Phi }=-\Sigma_{\hat{\zeta },\hat{\zeta }}\text{vec}\left(Q+K^{*T}RK^{*}\right).$
10:	Solve ***Z*** and *T* by Equation (12).
11:	Let *L* = K^*^Z + *T*, the optimal output regulation law is
	τ = −K^*^ζ + *L*.

## 4. Experimental results

### 4.1. Experimental setup

In the experiment, the control framework in Figure 3 is realized by the CPU PICO-WHU4 at a frequency of 1 k Hz. The rotation of the floating base is measured by the onboard IMU at a frequency of 400 Hz. The experimental data are saved at a frequency of 1 k Hz. The rotation of each actuated joint is measured by the motor encoder. The reference velocity and the reference height of the robot are set by the operator using a remote controller.

### 4.2. Training

**Figure 4:**
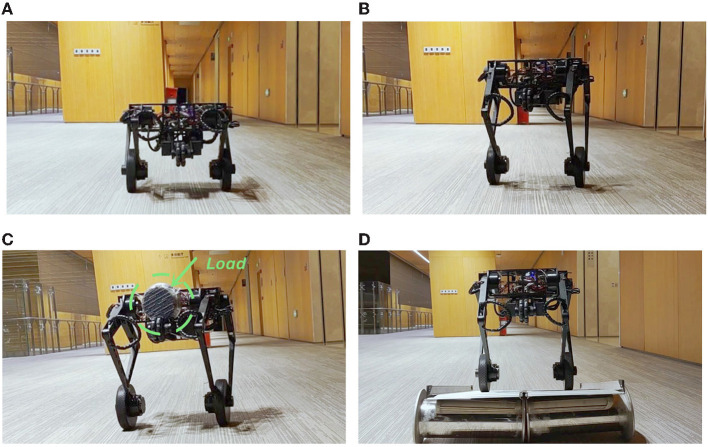
Images during the training. Many more maneuvers in action and experimental data are shown in the accompanying video. **(A)** Height = 0.33 m, **(B)** height = 0.5 m, **(C)** height = 0.5 m with the load, and **(D)** height = 0.5 m on the slope.

We train the data-driven optimal output regulation controller for Ollie in four scenarios to deal with uncertainties in model and disturbances. In our previous work (Cui et al., 2021; Zhang et al., 2022), the robot kept balanced, but it could not stand still, which was mentioned as a limitation. Hence, in the first two scenarios, we deal with the asymptotic stability problem in our previous works where the robot is at the height 0.33 m (Figure 4A) and the height 0.5 m (Figure 4B). Furthermore, we invoke two more challenging scenarios. In the third scenario, we mount an eccentric load of 3.5 kg in the front of the floating base (Figure 4C). As the floating base is about 11 kg, the heavy load would change the equilibrium point significantly. In the fourth scenario, we train the robot on a slope (Figure 4D). It is clear that extra torque is required to compensate for the component of gravity in the direction of the slope, which in turn implies that the equilibrium point changes. Notice that no information of the load (e.g., mass, shape, location) and the slope (e.g., gradient) is used in the training process, and they are treated as unknown disturbances.

During the training, the initial controller gain ***K***_0_ = [−74 − 26 − 10] is used, which is close to the resultant controller obtained from the VI training in Zhang et al. (2022). As the controller is stabilizing, the robot can move forward and backward repeatedly according to the remote control and the exploration noise β(*t*) = 1.6sin(8π*t*) + 0.8cos(12π*t*) is used to trigger more informative data. Training data of 6 s are collected for each experiment with the sampling interval 0.015 s. It means that, in Algorithm 1, *t*_0_ = 0, *t*_*s*_ = 6 s, and *t*_*k*+1_ − *t*_*k*_ = 0.015 s. Considering the sampling rate of 1 k Hz, there are 400 elements in each matrix in Equations (13) and (14). As suggested in Jiang and Jiang (2017), it is a good practice to guarantee the full rank condition in Alglorithm 1 by collecting data for which every matrix in Equations (13) and (14) has elements more than twice as many as the required rank. Hence, the full rank condition would be satisfied easily in this work.

**Figure 5:**
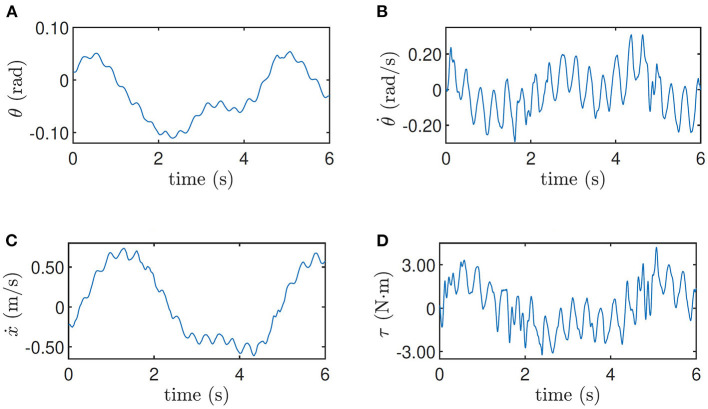
Training data for height = 0.33 m. **(A)** Pitch angle θ, **(B)** pitch velocity $\dot{\theta }$, **(C)** line velocity ẋ, and **(D)** input torque τ.

**Figure 6:**
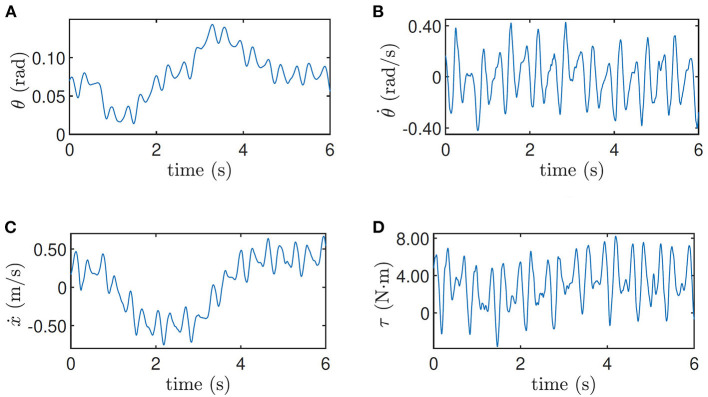
Training data for height = 0.5 m on the slope. **(A)** Pitch angle θ, **(B)** pitch velocity $\dot{\theta }$, **(C)** line velocity ẋ, and **(D)** input torque τ.

The training data when the robot is at the height 0.33 m and when the robot is at the height 0.5 m on the slope are shown in Figures 5, 6 respectively as an example, where the oscillation implies the exploration noise. Other training data are given in the accompanying video.

**Figure 7:**
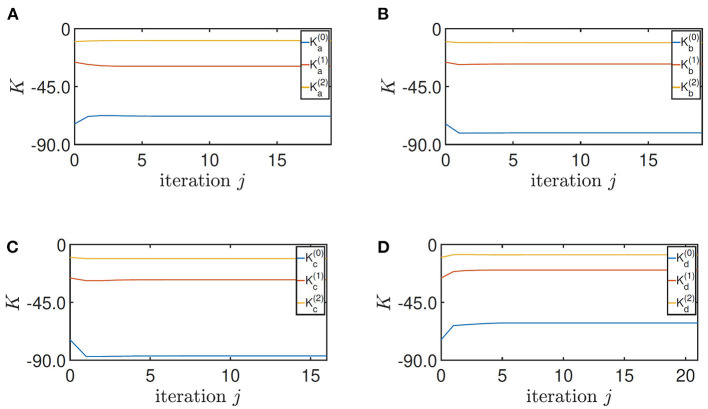
Convergence of the feedback gain *K*. **(A)** Height = 0.33 m, **(B)** height = 0.5 m, **(C)** height = 0.5 m with the load, and **(D)** height = 0.5 m on the slope.

Finally, by setting ***Q*** = diag(900, 400, 150), *R* = 1 in Algorithm 1, the control parameters of the four training converge to


(15) $$\begin{array}{l} \begin{array}{c} K_{a}^{*}=\left[-67.96-29.13-9.20\right],\;L_{a}=-4.21\\ K_{b}^{*}=\left[-80.92-27.38-10.79\right],\;L_{b}=-1.78\\ K_{c}^{*}=\left[-86.61-27.37-10.89\right],\;L_{c}=-5.97\\ K_{d}^{*}=\left[-61.04-19.86-7.97\right],\;L_{d}=1.30 \end{array} \end{array}$$

by the stopping criterion ϵ = 10^−4^ in 19, 18, 16, and 21 iterations, respectively (Figure 7). It should be highlighted that the fast convergence enables Algorithm 1 to be implemented online. Moreover, the robustness of the ADP-based algorithm guarantees the convergence to a small neighborhood of the optimal solution when noisy data are used (Pang et al., 2022).

The tuning of the parameter ***Q*** and *R* follows the standard process of LQR design. It is noticed in Zhang et al. (2022) and its accompanying video that the motor input is oscillating, which is caused by the overlarge regulation on the pitch angle of the robot. In this paper, the first diagonal term of ***Q*** is reduced accordingly. Moreover, by manual testing, the convergence of the algorithm is most sensitive on the third diagonal term of ***Q***, whose feasible range is from 90 to 280. In addition, the second diagonal term of ***Q*** should be larger than 180, and the first diagonal term can be any positive value. These results indicate the robustness of the convergence of Algorithm 1 on the parameters ***Q*** and *R*.

### 4.3. Testing

In the accompanying video, the trained controllers are updated to the robot online after the data collection. Here, we compare the initial controller and the result controller of AOOR directly to avoid the effect on the visual sense caused by the movement and exploration noises in the training process.

**Figure 8:**
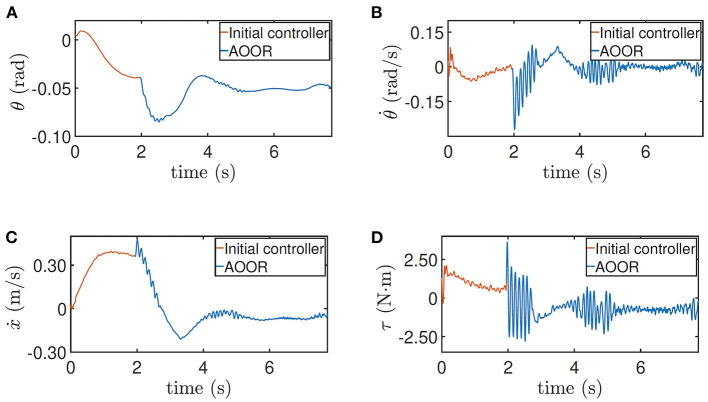
Testing results for height = 0.33 m. **(A)** Pitch angle θ, **(B)** pitch velocity $\dot{\theta }$, **(C)** line velocity ẋ, and **(D)** input torque τ.

**Figure 9:**
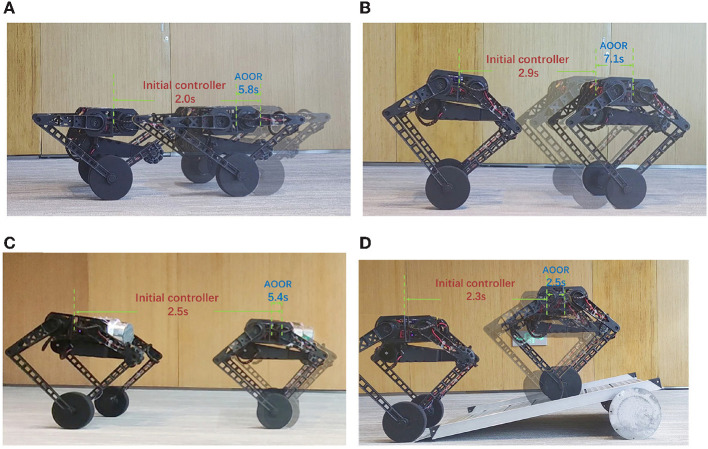
Still images during the testing. Many more maneuvers in action and experimental data are shown in the accompanying video. **(A)** Height = 0.33 m, **(B)** height = 0.5 m, **(C)** height = 0.5 m with the load, **(D)** height = 0.5 m on the slope.

In the first experiment, the robot is at the height 0.33 m, with experimental data given in Figure 8. The initial controller ***K***_0_ is used at first, and the experimenter uses the remote controller to force the robot to stand still. Then, after releasing the remote controller, the robot moves forward continuously in the first 2 s, which is indicated by the red line in Figure 8C. Then, by enabling the updated controller with $K_{a}^{*}$ and *L*_*a*_ at *t* = 2 s, the robot decelerates sharply and stays still in the following 6 s, which is indicated by the blue line in Figure 8C. In order to realize the fast regulation, the motor input is a little oscillating, but the effects on the pitch angle (Figure 8A) and the linear velocity (Figure 8C) are acceptable. The still image of the testing process is shown in Figure 9A. Combining the still image and the testing data, it is clear that the average velocity when using the AOOR is much slower than the initial case, reflected by the smaller displacement during a longer time. The small displacement during the AOOR period is caused by the convergence from the initial velocity to zero, which is naturally inevitable.

**Figure 10:**
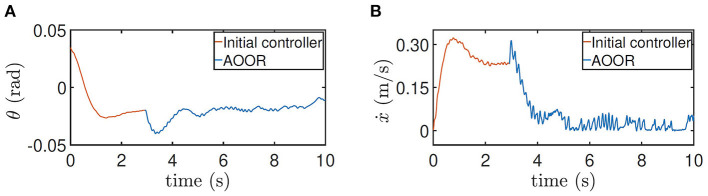
Testing results for height = 0.5 m. **(A)** Pitch angle θ. **(B)** line velocity ẋ.

**Figure 11:**
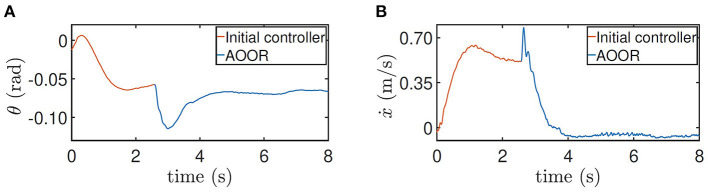
Testing results for height = 0.5 m with the load. **(A)** Pitch angle θ. **(B)** line velocity ẋ.

The testing processes when the robot is at the height 0.5 m and with the load are similar. By the testing data (Figures 10, 11), the robot decelerates and stands still (ẋ → 0) after the AOOR is enabled. The small displacement, including the deceleration phase, is demonstrated in Figures 9B, C respectively.

**Figure 12:**
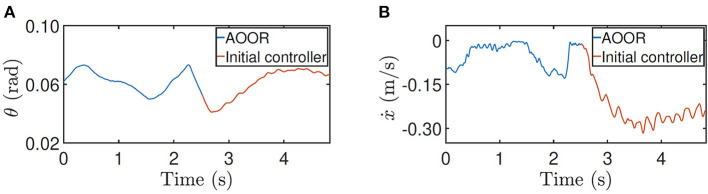
Testing results for height = 0.5 m on the slope. **(A)** Pitch angle θ. **(B)** line velocity ẋ.

For the testing on the slope, the method is similar. The difference is that the AOOR is used in the beginning to compare with the initial control latter. The reason is that, if the initial control is used in the beginning, the robot may leave the slope quickly before enabling the AOOR. The outstanding regulation performance is indicated by the data (Figure 12) together with the still image (Figure 9D).

The comparison of the controllers are summarized in Table 1. The much smaller displacement within the longer time by the AOOR indicates the strong regulation. The nonzero displacement includes the deceleration phase after enabling the AOOR, and any slight error in the regulation term *L* may also cause a small displacement in the steady state.

**Table 1:** Comparison of the controllers during the testing.

**Scenario**	**Initial controller**		**AOOR**	
	**Time**	**Displacement**	**Time**	**Displacement**
	**(s)**	**(m)**	**(s)**	**(m)**
No. 1	2.0	0.46	5.8	0.15
No. 2	2.9	0.56	7.1	0.15
No. 3	2.5	1.01	5.4	0.04
No. 4	2.3	0.42	2.5	0.10

### 4.4. Application

The AOOR is applied when the robot is balancing a rolling cylindrical water bottle on the top. A PD controller is used to tune the floating base by the feedback of the bottle position (*s*_*b*_) and velocity (ṡ_*b*_) with respect to the center of the floating base in the x direction by the following laws


$$\begin{array}{l} {\ddot{q}}_{\left(5\right)}^{\text{des}}=-k_{p}^{\text{ball}}s_{b}-k_{d}^{\text{ball}}{ṡ}_{b}+k_{p}^{\text{base}}q_{\left(5\right)}+k_{d}^{\text{base}}{\dot{q}}_{\left(5\right)}, \end{array}$$

where ***q***_(5)_ denotes the pitch angle of the floating base of the robot. Hence, the controller aims to regulate the ball at the target position ($s_{b}^{*}=0$) with the PD gain $k_{p}^{\text{ball}}$, $k_{d}^{\text{ball}}$, and to keep the floating base horizontal (***q***_(5)_ = 0) wit the PD gain $k_{p}^{\text{base}}$, $k_{d}^{\text{base}}$. The signs depend on the positive directions of the predefined coordinates. In addition, the state *s*_*b*_ is updated by a self-developed tactile sensor (Zhao et al., 2022) by Tencent Robotics X.

**Figure 13:**
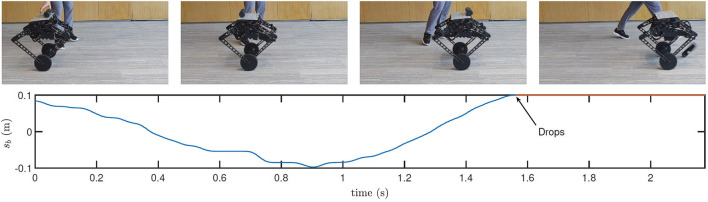
Still images and real-time data obtained during bottle balancing using the initial controller. Many more maneuvers in action and experimental data are shown in the accompanying video.

First, with the initial controller, the robot keeps moving forward as shown in Figure 13. In consequence, when the bottle is placed on the top, the overlarge relative velocity between the robot and the bottle is a destructive initial condition for the bottle balancing task. As shown in the figure, the control is not converging, and the bottle drops in 2 s.

**Figure 14:**
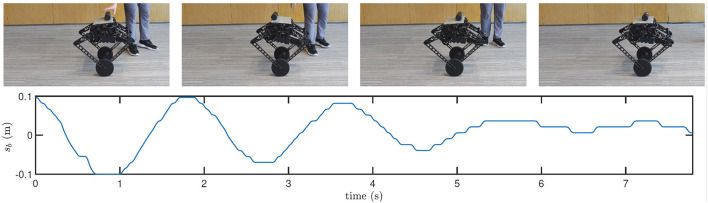
Still images and real-time data obtained during bottle balancing using the AOOR. Many more maneuvers in action and experimental data are shown in the accompanying video.

Next, with the AOOR, the robot almost stands still, so the ignorable relative velocity provides an ideal initial condition for the bottle balancing task. As shown in Figure 14, the bottle is controlled to the middle of the top. Combining with the mobility over complex terrains, this result shows the possibility to apply the wheel-legged robot to deliver goods for which the contact with the robot is not closed.

## 5. Conclusion

The mismatch between the ideal modeling in simulation and the real robot would cause the deterioration of the control performance. This is reflected in the experiments of our previous works, where the robot is balanced but cannot stand still without extra manual adjustment. Moreover, it is also troublesome to deal with the possible variations in the robot dynamics during the operation caused by both internal and external factors. To solve these problems, the idea of AOOR in the author's previous publication is applied in the balance controller design of the wheel-legged robot Ollie. In the experiments, by a training process of 6 s using the AOOR, the optimality of the controller is preserved when the working condition changes. In addition, the noticed problems in the previous works are solved, where the robot can stand still when it is at different heights. Furthermore, more challenging experiments are designed to test the AOOR-based controller by adding an eccentric heavy load to the robot, and by driving the robot on a slope. Finally, the proposed AOOR-based controller improves the success rate when the robot is balancing a cylinder water bottle on the top cover. This result makes it possible to apply the robot to deliver goods for which the limitation on the shape is relaxed.

Several feasible future studies would be interesting to further improve the control performance. In this paper, the working condition of the robot is changed during operation but is not time-varying. In the case of time-varying disturbances, it is possible to extend the AOOR algorithm to include the output regulation term that deals with time-varying disturbances. Moreover, in our previous research, the robot could be stabilized from arbitrary (unstable) input by the VI algorithm. Hence, it is also interesting to explore a VI-based AOOR algorithm to regulate the robot to its equilibrium point from arbitrary input. Finally, the robot works in its linearizable region in this work. However, for a wider working range or for more complex robotic systems, it is worth studying to extend the AOOR to nonlinear systems by combining the nonlinear ADP and nonlinear output regulation, where the computational efficiency would be a core issue.

## Data availability statement

The original contributions presented in the study are included in the article/Supplementary material, further inquiries can be directed to the corresponding author.

## Author contributions

JZ and ZL proposed the theory, conducted the experiment, and wrote the manuscript. SW proposed the general idea of this theory and its application to the robot. SW and YZ supervised this work and revised the manuscript. YD and JT designed and fabricated the tactile sensor on the robot. RZ designed the data collection board of the tactile sensor. JL and KC designed and integrated the hardware and embedded system of the robot. DZ designed and maintained the mechanical system of the robot. JH revised the manuscript. WG discussed the theory and revised the manuscript. All authors contributed to the article and approved the submitted version.
